# Author Correction: Integrative spatial and genomic analysis of tumor heterogeneity with Tumoroscope

**DOI:** 10.1038/s41467-025-58177-8

**Published:** 2025-03-21

**Authors:** Shadi Shafighi, Agnieszka Geras, Barbara Jurzysta, Alireza Sahaf Naeini, Igor Filipiuk, Alicja Rączkowska, Hosein Toosi, Łukasz Koperski, Kim Thrane, Camilla Engblom, Jeff E. Mold, Xinsong Chen, Johan Hartman, Dominika Nowis, Alessandra Carbone, Jens Lagergren, Ewa Szczurek

**Affiliations:** 1 Faculty of Mathematics, Informatics and Mechanics, University of Warsaw, Warsaw, Poland; 2 Sorbonne Universite, CNRS, IBPS, Laboratoire de Biologie Computationnelle et Quantitative, Paris, France; 3 Cancer Research UK Cambridge Institute, Cambridge, UK; 4 Faculty of Mathematics and Information Science, Warsaw University of Technology, Warsaw, Poland; 5 Department of Statistics, Columbia University, New York, NY 10027 USA; 6 Irving Institute for Cancer Dynamics, Columbia University, New York, NY 10027 USA; 7 SciLifeLab, School of EECS, KTH Royal Institute of Technology, Stockholm, Sweden; 8 Department of Pathology, Medical University of Warsaw, Warsaw, Poland; 9 Department of Gene Technology, KTH Royal Institute of Technology, SciLifeLab, Stockholm Sweden; 10 Department of Cell and Molecular Biology, Karolinska Institutet, Solna, Sweden; 11 SciLifeLab, Department of Medicine Solna, Center of Molecular Medicine, Karolinska Institute and University Hospital, Stockholm, Sweden; 12 Department of Oncology-Pathology, Karolinska Institutet, Stockholm, Sweden; 13 Department of Clinical Pathology and Cancer Diagnostics, Karolinska University Hospital, Stockholm, Sweden; 14 Laboratory of Experimental Medicine, Medical University of Warsaw, Warsaw, Poland; 15 Institut Universitaire de France, Paris, France; 16 Institute of AI for Health, Helmholtz Munich, German Research Center for Environmental Health, Neuherberg, Germany

**Keywords:** Tumour heterogeneity, Statistical methods, Cancer genomics

Correction to: *Nature Communications* 10.1038/s41467-024-53374-3, published online 29 October 2024

In this article the authors name Alicja Rączkowska was incorrectly given as Alicja Ra̧czkowska. The original article has been corrected.

