# Supplementary material for: Integrative spatial and genomic analysis of tumor heterogeneity with Tumoroscope
Source: Nat Commun. 2024 Oct 29;15:9343. doi: 10.1038/s41467-024-53374-3 (PMC11522407; doi:10.1038/s41467-024-53374-3)
Supplement: Supplementary file 1 — Supplementary Information [file 41467_2024_53374_MOESM1_ESM.pdf]

Integrative Spatial and Genomic Analysis of Tumor Heterogeneity with Tumoroscope Supplementary Information

Shadi Shafighi[1,2,3], Agnieszka Geras[1,4,5,6], Barbara Jurzysta[1], Alireza Sahaf Naeini[1], Igor Filipiuk[1], Alicja Rączkowska[1], Hosein Toosi[7], Łukasz Koperski[8], Kim Thrane[9], Camilla Engblom[10,11], Jeff E Mold[10], Xinsong Chen[12], Johan Hartman[12,13], Dominika Nowis[14], Alessandra Carbone[2,15], Jens Lagergren[7,#], and Ewa Szczurek[1,16*,#]

[1] *Faculty of Mathematics, Informatics and Mechanics, University of Warsaw, Warsaw, Poland*

[2] *Sorbonne Universite, CNRS, IBPS, Laboratoire de Biologie Computationnelle et Quantitative, Paris, France*

[3] *Cancer Research UK Cambridge Institute, Cambridge, UK*

[4] *Faculty of Mathematics and Information Science, Warsaw University of Technology, Warsaw, Poland*

[5] *Department of Statistics, Columbia University, New York, NY, 10027, USA*

[6] *Irving Institute for Cancer Dynamics, Columbia University, New York, NY, 10027, USA*

[7] *SciLifeLab, School of EECS, KTH Royal Institute of Technology, Stockholm, Sweden*

[8] *Department of Pathology, Medical University of Warsaw, Warsaw, Poland*

[9] *Department of Gene Technology, KTH Royal Institute of Technology, SciLifeLab, Stockholm, Sweden*

[10] *Department of Cell and Molecular Biology, Karolinska Institutet, Solna, Sweden*

[11] *SciLifeLab, Department of Medicine Solna, Center of Molecular Medicine, Karolinska Institute and University Hospital, Stockholm, Sweden*

[12] *Department of Oncology-Pathology, Karolinska Institutet, Stockholm, Sweden*

[13] *Department of Clinical Pathology and Cancer Diagnostics, Karolinska University Hospital, Stockholm, Sweden*

[14] *Laboratory of Experimental Medicine, Medical University of Warsaw, Warsaw, Poland*

[15] *Institut Universitaire de France, Paris, France*

[16] *Institute of AI for Health, Helmholtz Munich, German Research Center for Environmental Health, Neuherberg, Germany*

[*] *Correspondence: szczurek@mimuw.edu.pl*

[#] *These authors have contributed equally to this work*

Supplementary Table

Property	Simulation	Prostate dataset	Breast dataset
number of the clones in the evolutionary tree	5	4	7
number of mutations in the genotype	30	282	608
average clone per spot	1, 2.5, 4.5	-	-
average number of mutations per clone	5.1 (16.9%), 13.6 (45.3%),15 (50%)	110 (39%)	272.1 (44.7%)
average number of reads present in each spot	18, 50, 80, 110	79.5	17.6
average noise introduced to the number of cells	0,1,10	-	-

Supplementary Table 1: The setups used for simulation and the real values for prostate and breast cancer datasets.

Supplementary Figures

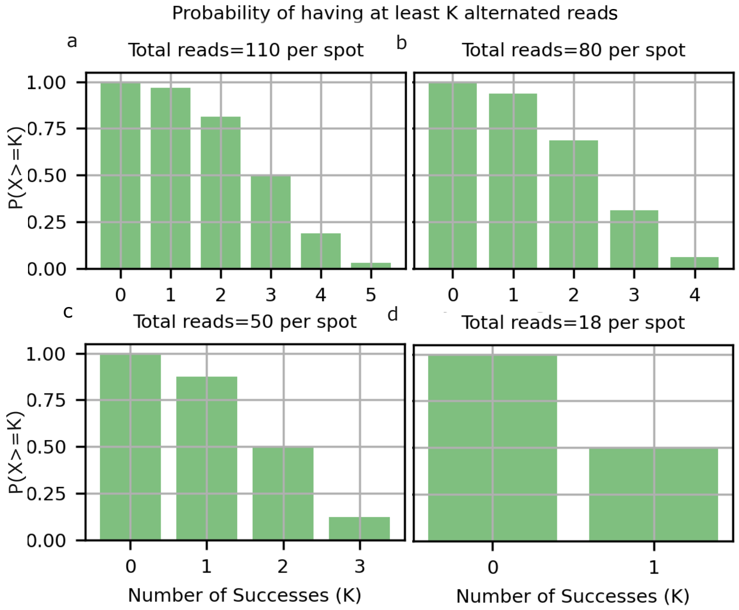

Supplementary Figure 1: Probability of detecting alternated reads in various simulation configurations. To calculate the probability of observing alternated reads, we consider four different total read counts 110 (panel **a**), 80 (**b**), 50 (**c**), and 18 (**d**) per spot (assumed as the mean total counts in our simulation configurations in Figure 2). For each configuration, we divide the total number of reads per spot by the mean number of cells in each spot (assumed to be 25, 25, 25, 25, respectively, in the simulation configurations), obtaining the total numbers of reads per cell: around 5, 4, 3, 1, respectively. The probability of observing at least K reads (successes; x-axis) out of 5, 4, 3, reads or 1 read is determined by 1 minus the cumulative distribution function (cdf) for the binomial distribution of alternated read counts, denoted as 1-cdf(K) (y-axis).

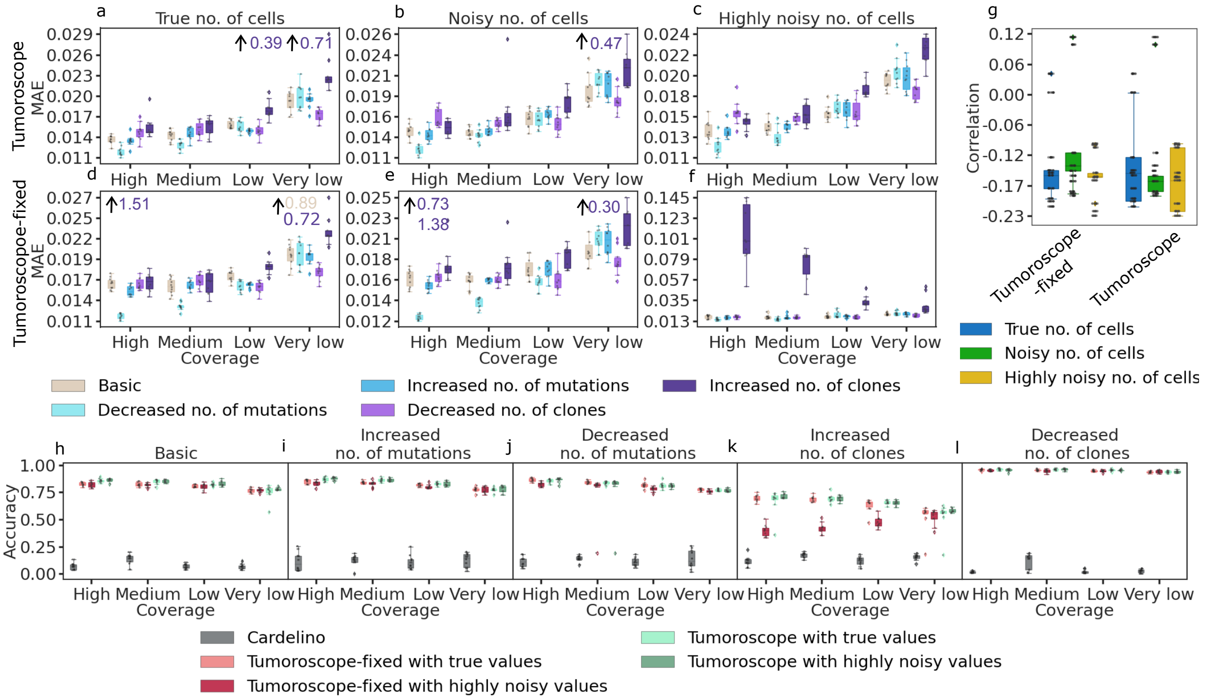

Supplementary Figure 2: Performance of Tumoroscope on simulated data with a high number of reads. **a-c** Mean Average Error (MAE; y-axis) as a function of spot coverage (x-axis) in different simulation setups (colors) for Tumoroscope, for different noise levels in the cell count provided at input: no noise (**a**), medium noise (**b**) and high noise (**c**). **d-f** The same as in (**a-c**), but for Tumoroscope-fixed. **g** Correlation (y-axis) between the average spot coverage and the average error in all the setups is negative for both model versions (x-axis), regardless of the noise in the number of cells provided at the input (colors). **h-l** Comparison of the accuracy (y-axis) of the model between cardelino (gray) and two versions of the model given true and highly noisy values for the number of cells (colors), depending on the spot coverage (x-axis), in different simulation setups: basic (**h**), increased (**i**) and decreased (**j**) number of mutations, increased (**k**) and decreased (**l**) number of clones. Here, high, medium, low, and very low are corresponding to the average number of reads present in each spot of 2246, 1488, 734, and 297 respectively. Overall, compared to Fig. 2, having a higher number of reads increased the performance (strongly decreased MAE) for the estimation of the fraction of the clones in spots. **f** In the case when Tumoroscope-fixed is given a fixed number of cells that is highly noisy, increasing the number of clones in spots entangles the deconvolution problem. Consequently, for Tumoroscope-fixed, the highly noisy input confounds the model the most when the read counts are high and the model cannot assign the right clones to the spots, resulting in the largest MAE. In each panel, diamonds indicate outliers. The boxplots in panels (a-f) and (h-l) are based on 10 data points each, corresponding to n=10 generated datasets for each setup. In panel (g), each boxplot represents 20 data points, corresponding to Pearson correlations, calculated across the 10 datasets for the n=20 different setups.

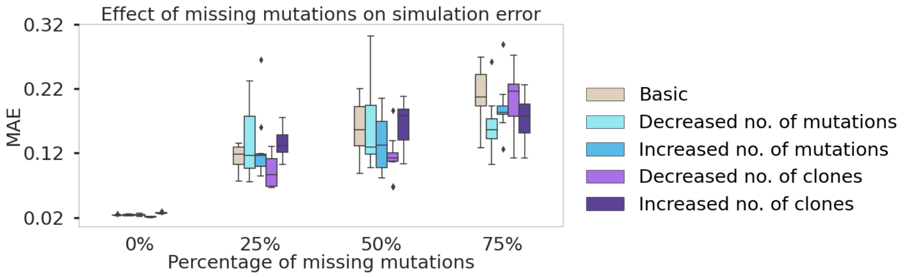

Supplementary Figure 3: Assessing Tumoroscope performance with partial mutation observations for different simulation setups (colors). In each panel, diamonds indicate outliers. The boxplots are based on 10 data points each, corresponding to n=10 generated datasets for each setup.

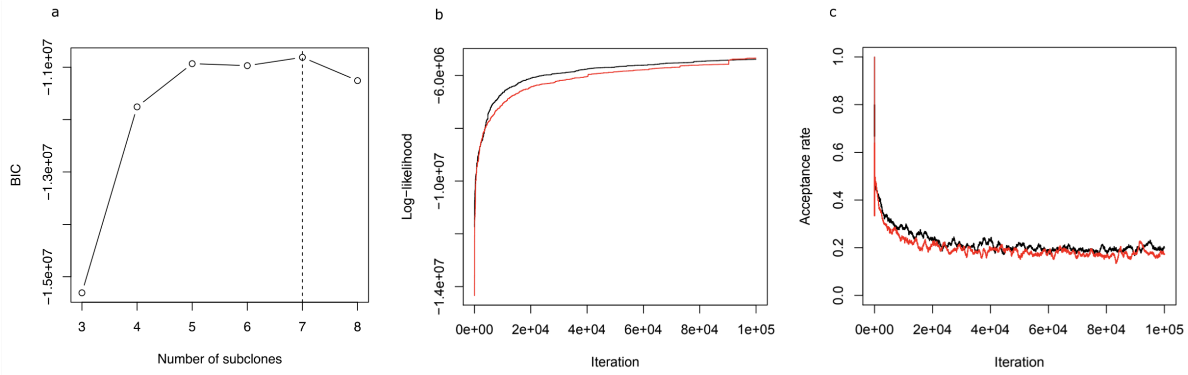

Supplementary Figure 4: Analysis of the Canopy tree inference for the breast cancer dataset. **a** Bayesian Information Criterion (BIC; y-axis) of the Canopy model for different numbers of clones in the tree (x-axis). We selected the tree with seven clones, for which the BIC was the largest (indicated with the dotted vertical line). **b** Log-Likelihood of two MCMC chains of Canopy (y-axis) across MCMC iterations (x-axis), showing the convergence of the MCMC procedure. **c** Acceptance rate (y-axis) across iterations (x-axis). The acceptance rate converges to around the desired value of around 0.2.

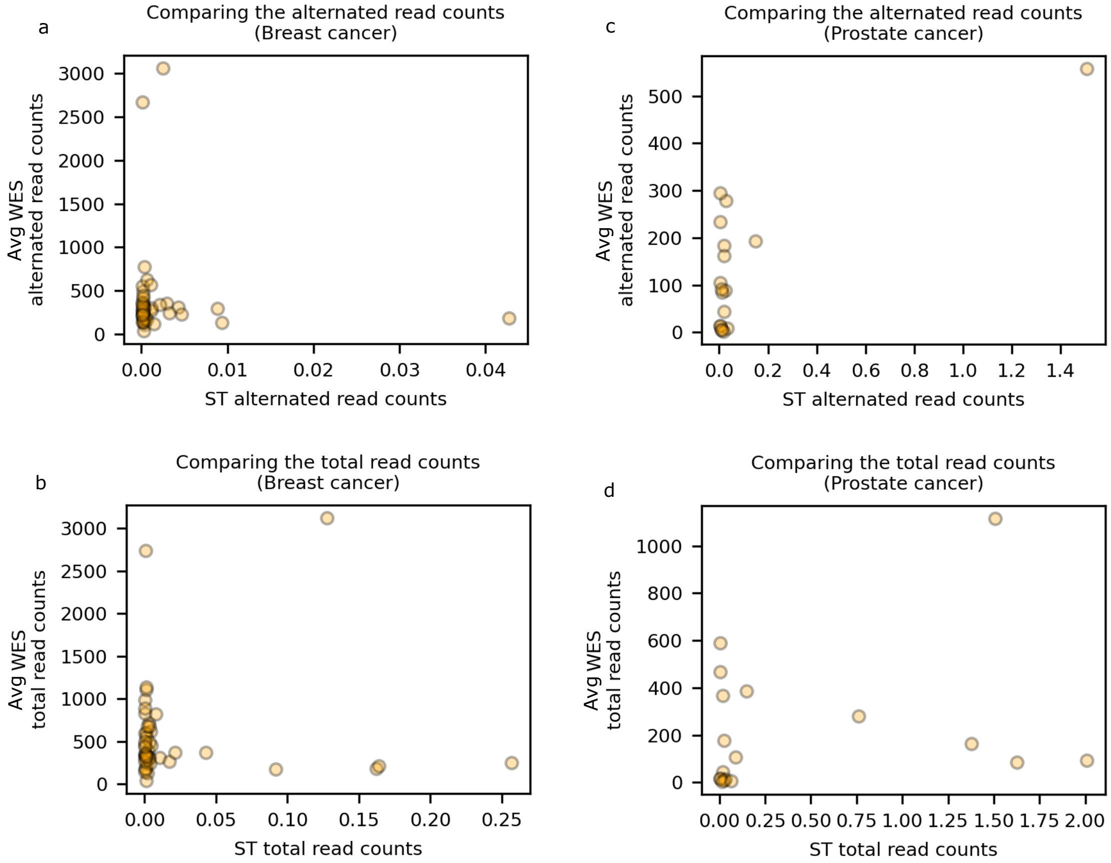

Supplementary Figure 5: Comparison of alternated and total read counts between WES and ST in breast (a-b) and prostate (c-d) cancer data. Each point is representing a mutation. For breast cancer, we show data for n=46 mutations, and for prostate cancer, n=18 mutations. X-axis: the average read counts per spot for each mutation. Y-axis: the average read counts per sample in WES for the same mutations.

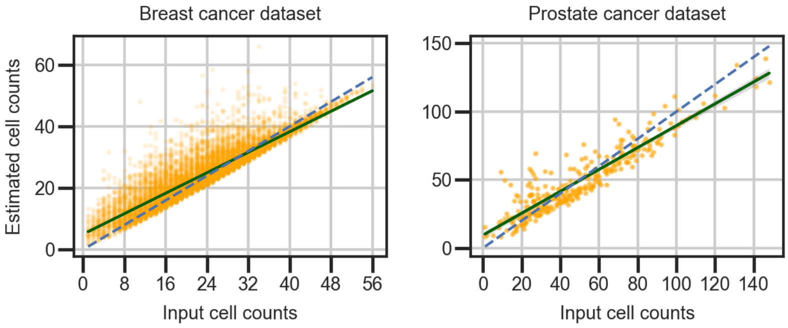

Supplementary Figure 6: Comparision of the input (retrieved from H&E slides) and the inferred cell count of the spots in both breast and prostate cancer datasets. Each point represents a single spot, with n=11,461 points for the breast cancer dataset and n=294 points for the prostate cancer dataset. The dark green line corresponds to the regression line, and the dashed line is the x=y line, indicating points for which the input and estimated counts would be the same.

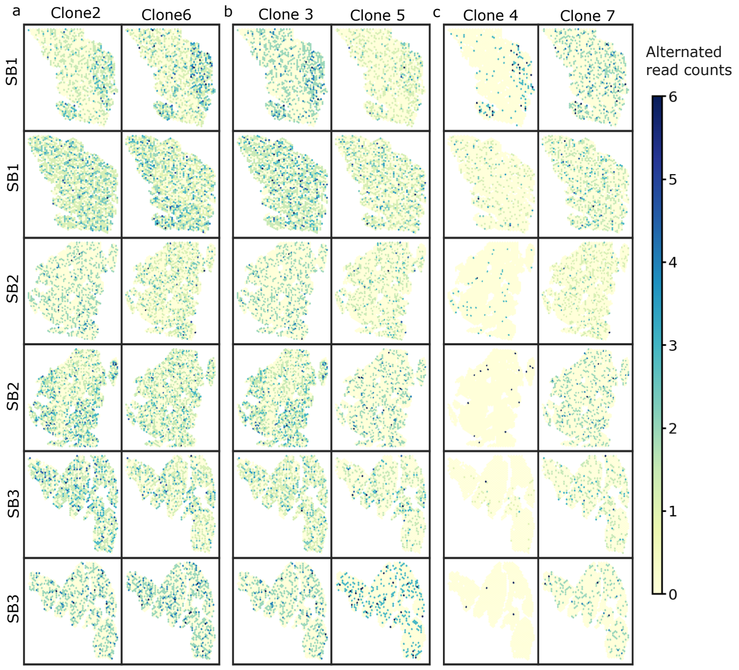

Supplementary Figure 7: Maps of alternated read counts over the mutations specific to each clone. **a** The alternate read counts corresponding to mutations found in clone 2 but not in clone 6, and conversely, mutations present in clone 6 but absent in clone 2. **b** The alternate read counts corresponding to mutations found in clone 3 but not in clone 5, and conversely, mutations present in clone 5 but absent in clone 3. **d** The alternate read counts corresponding to mutations found in clone 4 but not in clone 7, and conversely, mutations present in clone 7 but absent in clone 4. Rows: sections of the breast cancer tissue with two nearby samples per section, SB1, SB2, and SB3.

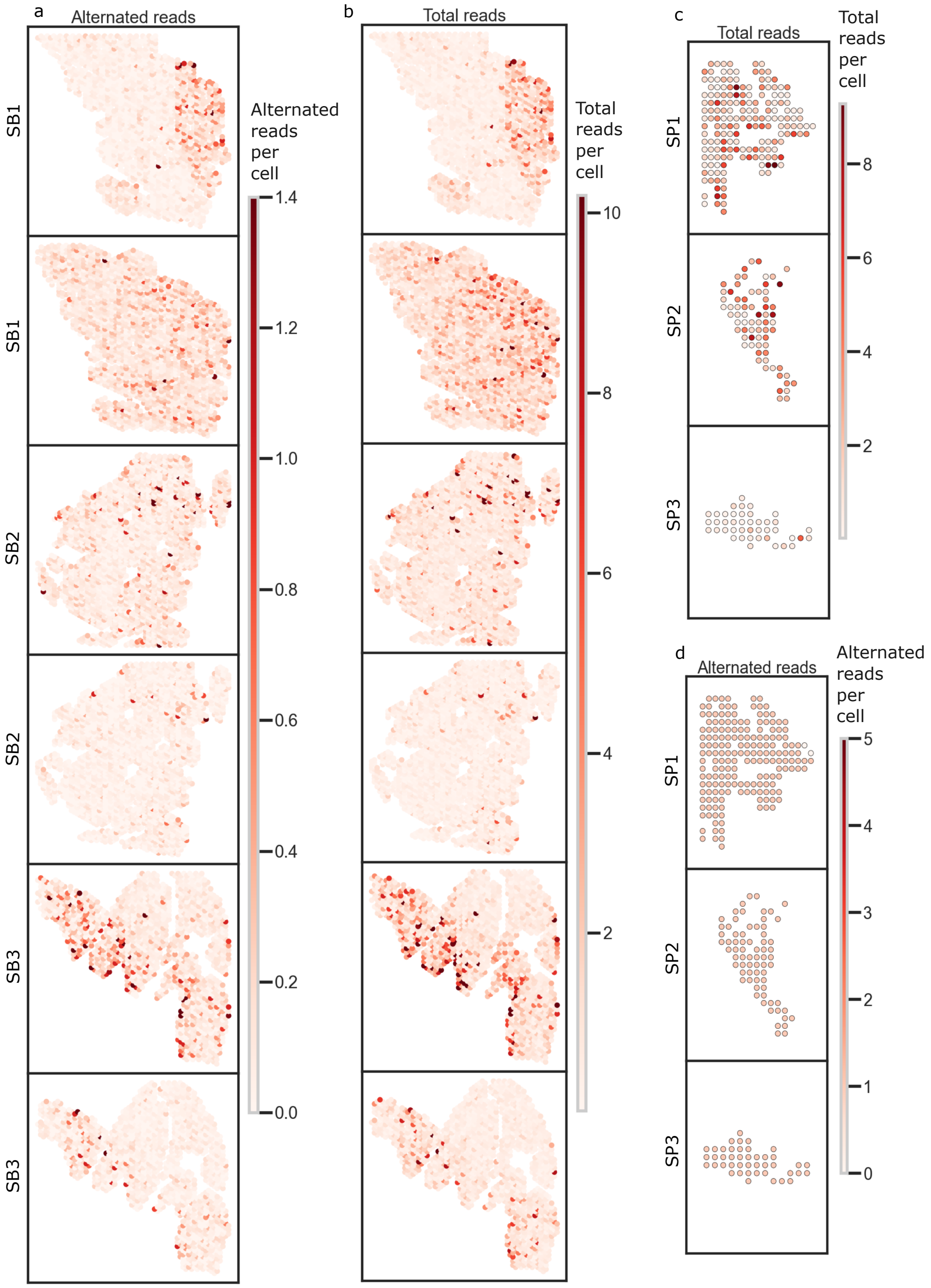

Supplementary Figure 8: Alternated and total read counts per cell over all the selected mutations in breast (a-b) and prostate (c-d) cancer data. While it is expected that spot coverage varies due to technical artifacts and the activity of different cancer clones, our findings demonstrate the presence of mutated reads across all tissues, affirming their cancerous nature. Rows in **a**,**b**: sections of the breast cancer tissue with two nearby samples per section, SB1, SB2, and SB3. Rows in **c**, **d**: sections of the prostate cancer tissue, SP1, SP2, and SP3. The value of each spot is calculated by dividing the number of reads observed within that spot by the number of cells it contains. The values shown in **c**, **d** are after the removal of outliers exceeding 10 (2 outliers for **c** and 6 for **d**).

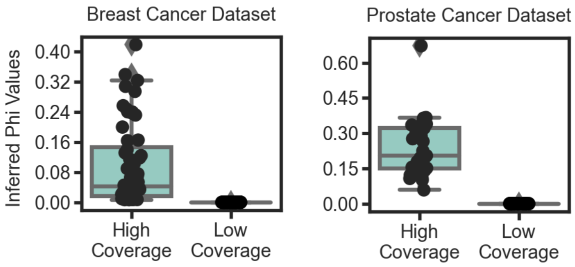

Supplementary Figure 9: Inferred per variant per cell expression values agree with the observed coverage in the data. $\mathbf{\Phi}_i$ values for the mutation i which belongs to the subset of the mutations with 10% of the highest (High Coverage) and the lowest (Low Coverage) total read count, where $\mathbf{\Phi}_i = \sum_k \mathbf{\Phi}_{i,k}$ (the sum of inferred mutation coverage levels for variant i over all clones), for breast cancer (left) and prostate cancer (right) data. In each panel, diamonds correspond to outliers. Each point represents a single mutation, with n=60 points for the breast cancer dataset and n=28 points for the prostate cancer dataset for each of the boxplots.

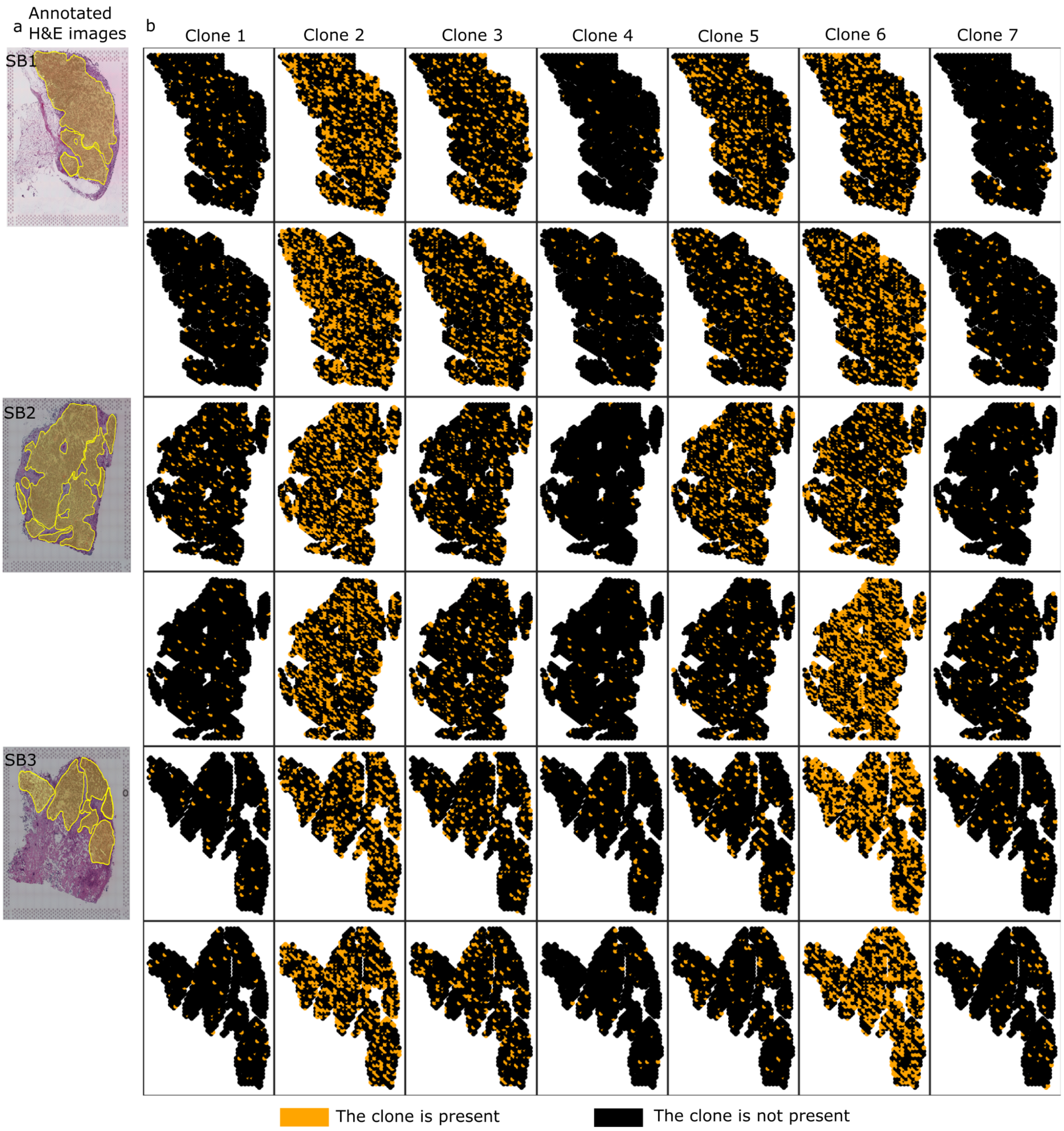

Supplementary Figure 10: Spatial arrangement of cancer clones identified by cardelino for the breast cancer dataset. **a** Pathologist's annotations of the cancerous areas on the H&E images for sections SB1, SB2, and SB3. **b** For each section, two rows correspond to the two nearby samples and seven columns correspond to the presence of the clone in the spots inferred by cardelino.

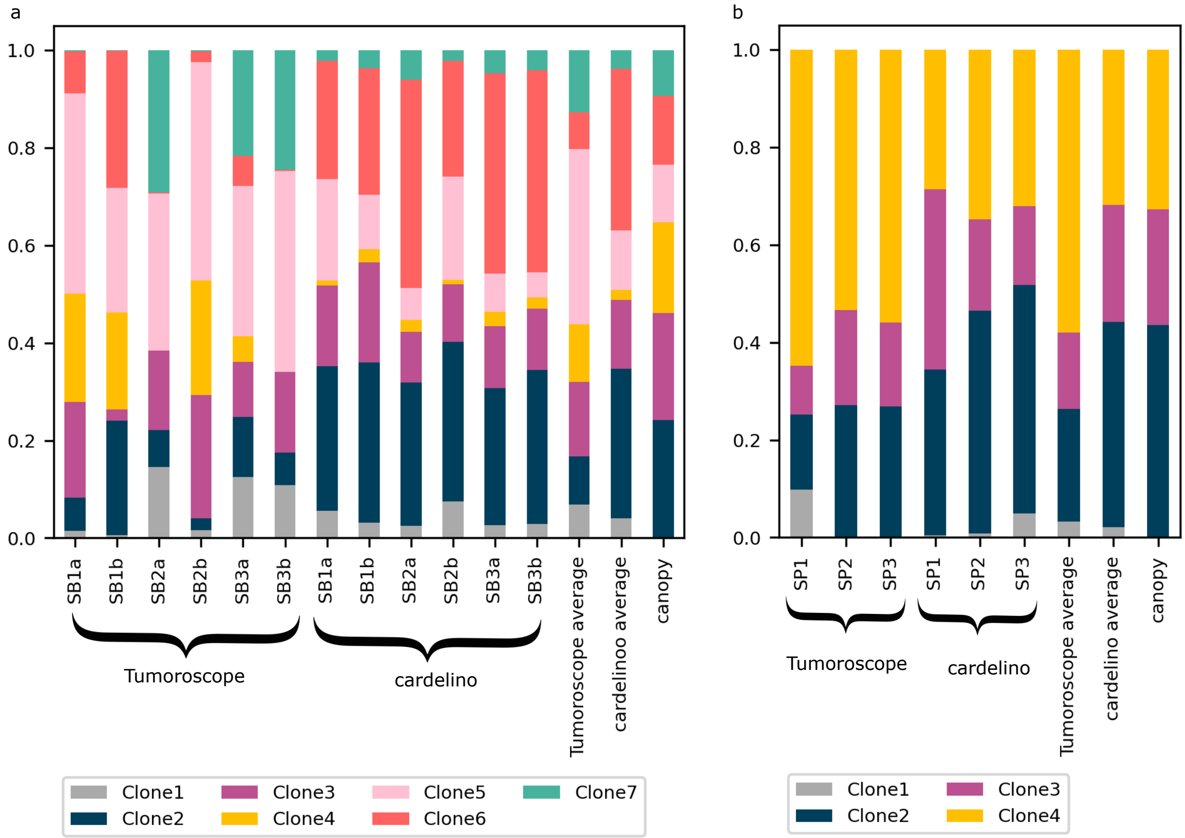

Supplementary Figure 11: Proportion of each clone in each section. a Proportions of inferred clones by Tumoroscope and cardelino for the breast cancer dataset. The proportions were computed by summing the inferred fractions across spots for each ST section. Averages over sections and clone frequences inferred by Canopy from bulk DNAseq data are also shown. **b** Proportions of inferred clones by Tumoroscope and cardelino for the prostate cancer dataset.

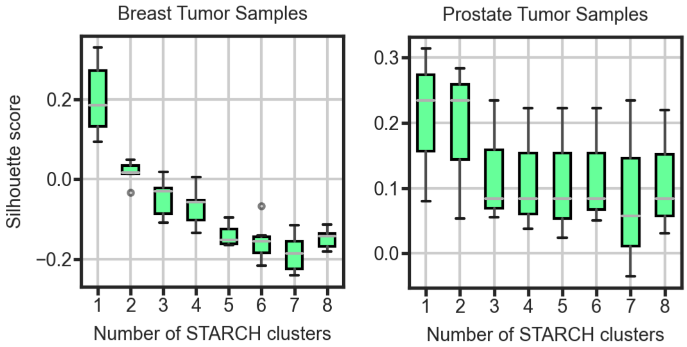

Supplementary Figure 12: Silhouette score of the STARCH clustering for 1 to 8 clusters. In both breast and prostate tumor samples, clustering the spots to one cancerous and one normal cluster got the highest score. In each panel, dots correspond to outliers. Each boxplot for the breast cancer data represents 6 values from n=6 samples, while each boxplot for the prostate cancer data represents 3 values from n=3 samples.

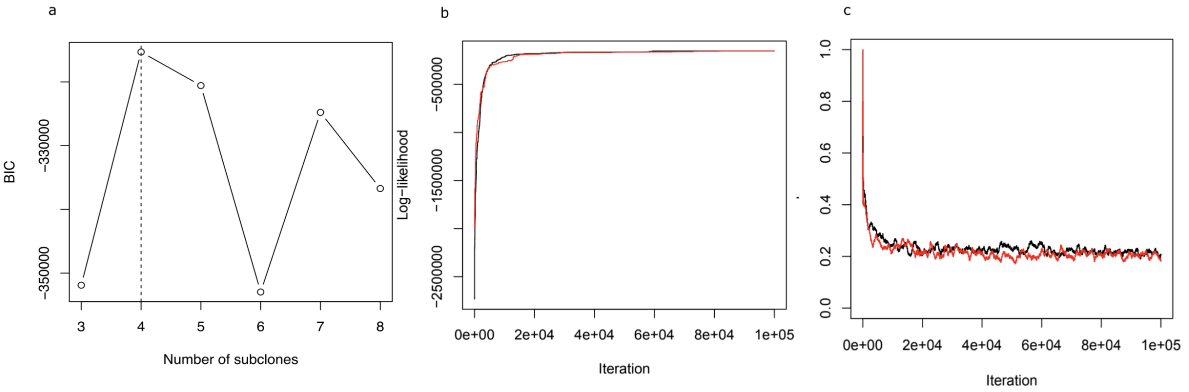

Supplementary Figure 13: Analysis of the Canopy tree inference for prostate cancer dataset. **a** Bayesian Information Criterion (BIC; y-axis) of the Canopy model for different numbers of clones in the tree (x-axis). We selected the tree with four clones, for which the BIC was the largest (indicated with the dotted vertical line). **b** Log-Likelihood of two MCMC chains of Canopy (y-axis) across MCMC iterations (x-axis), showing the convergence of the MCMC procedure. **c** Acceptance rate (y-axis) across iterations (x-axis). The acceptance rate converges to around the desired value of around 0.2.

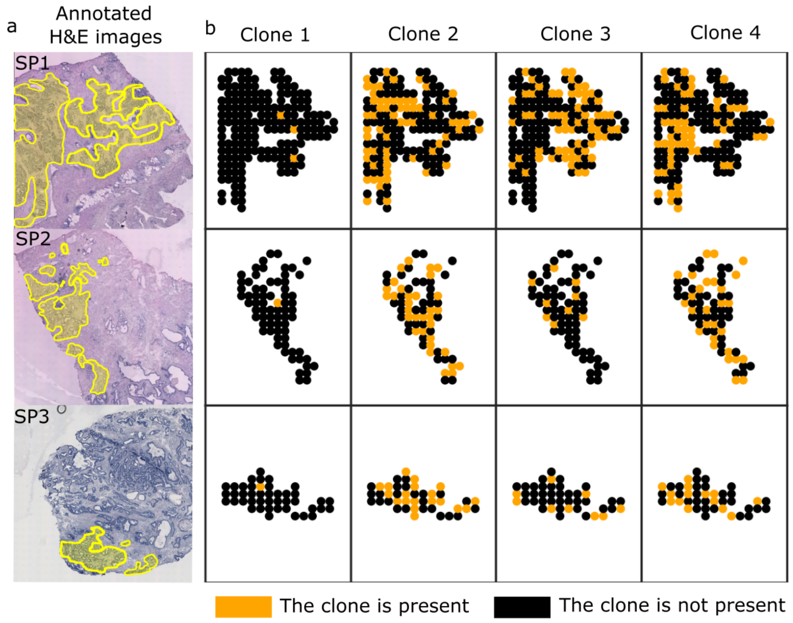

Supplementary Figure 14: Results obtained by cardelino for the prostate cancer dataset. **a** Pathologist's annotation of the cancerous areas on the H&E images for sections SP1, SP2, and SP3. **b** For each section (rows), there are four columns corresponding to the presence of the clones in the spots.

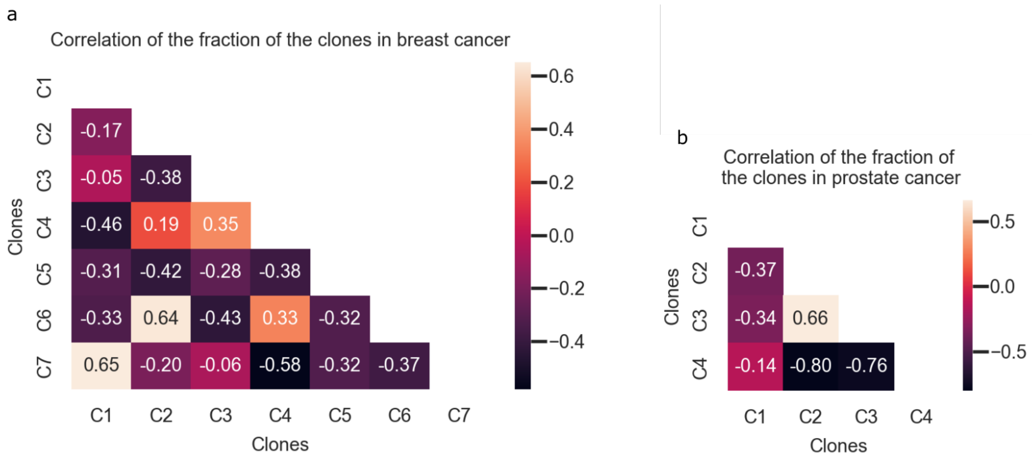

Supplementary Figure 15: Pairwise Pearson correlations of the proportions of all the spots taken by the clones for breast cancer (**a**) and for prostate cancer (**b**) data.

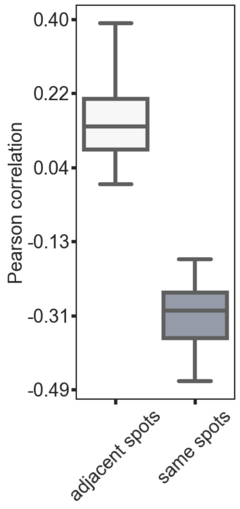

Supplementary Figure 16: Distribution of the Pearson correlation between the proportions of the spots taken by the clones 3 and 5. The correlation between proportions of clones 5 and 3 in adjacent spots was computed for 20 different sets of randomly sampled pairs of 100 adjacent spots. The correlation between the proportions of clones 5 and 3 in the same spots was computed for n=20 sets of 100 randomly sampled spots.

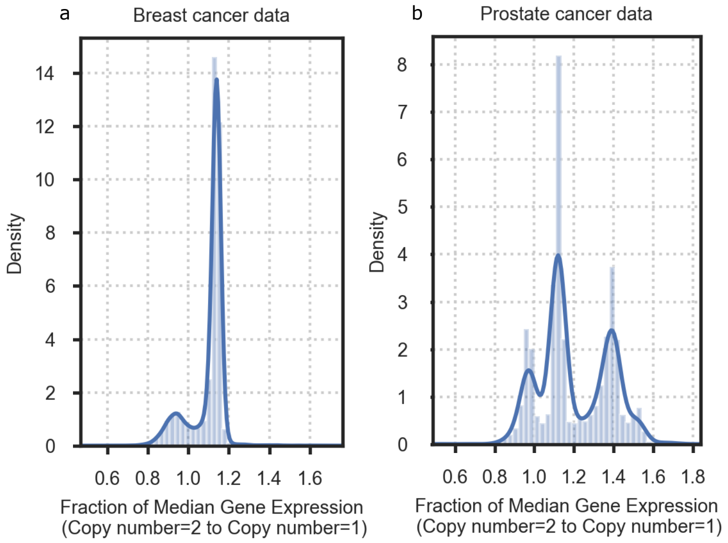

Supplementary Figure 17: Agreement of gene expression values computed based on Tumoroscope results with copy numbers inferred by STARCH for breast and prostate cancer data. For each gene, we determined its predicted expression in individual spots of spatial transcriptomics data through the application of a fitted regression model (Fig. 1 h). This involved computing the expected gene expression values based on the clone-specific gene expression inferred by the regression model for each clone, the fraction of clones in each spot as determined by Tumoroscope, and the number of cells in each spot. Subsequently, the copy numbers for genes in each spot were determined using STARCH. We then calculated the average predicted expression in spots with copy number 2 and copy number 1 for each gene (n=9799 genes for breast cancer data and n=8559 genes for prostate cancer data). The resulting distribution (y-axis) over the fraction of these two values (x-axis) shows that the predicted gene expression for genes with copy number 2 tends to be higher than for the genes with copy number 1, for both breast (a) and prostate cancer (b).

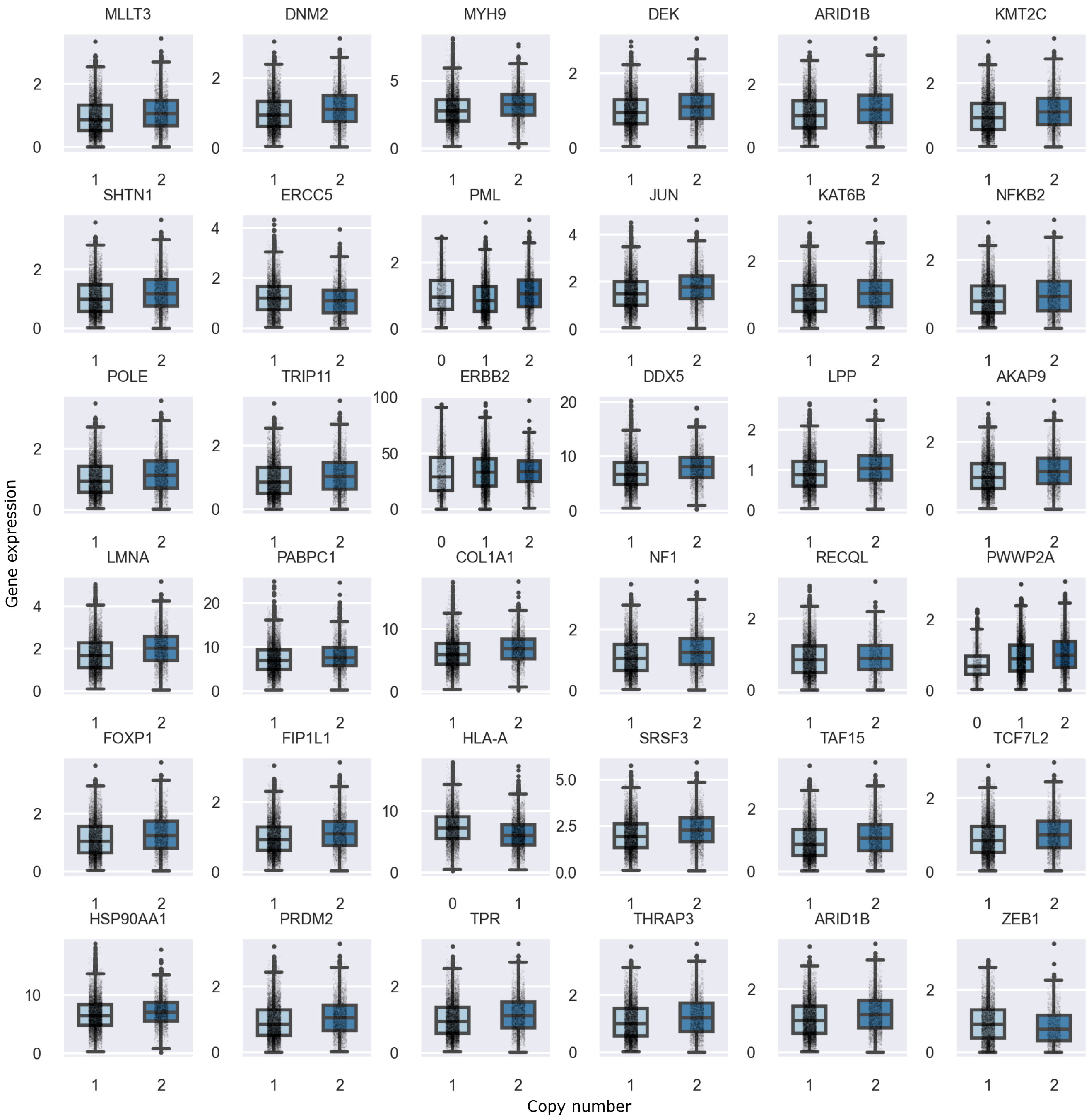

Supplementary Figure 18: Agreement of gene expression values computed based on Tumoroscope results with copy numbers inferred by STARCH for breast cancer data. For each gene, we determined its predicted expression in individual spots of spatial transcriptomics data through the application of a fitted regression model (Fig. 1 h). This involved computing the expected gene expression values based on the clone-specific gene expression inferred by the regression model for each clone, the fraction of clones in each spot as determined by Tumoroscope, and the number of cells in each spot. Subsequently, we analyzed gene copy numbers across tumor samples using STARCH. Focusing on mutated genes appearing in the evolutionary tree, we selected genes that were detected across a minimum of 20 tumor spots for both copy number value 1 and 2. This filtering ensured we had sufficient sample sizes for comparison. We then plotted the distribution of expression levels (y-axis) grouped by copy number value (x-axis) for these selected genes. The resulting distributions show that genes tending to have higher copy numbers in a tumor sample also exhibit elevated expression compared to genes with lower copy numbers in the same sample.

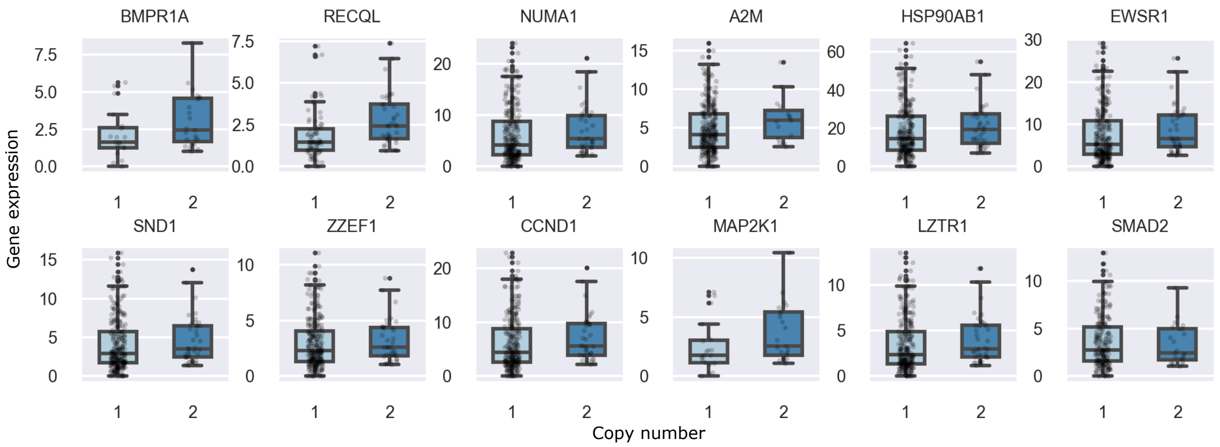

Supplementary Figure 19: Agreement of gene expression values computed based on Tumoroscope results with copy numbers inferred by STARCH for prostate cancer data. For each gene, we determined its predicted expression in individual spots of spatial transcriptomics data through the application of a fitted regression model (Fig. 1 h). This involved computing the expected gene expression values based on the clone-specific gene expression inferred by the regression model for each clone, the fraction of clones in each spot as determined by Tumoroscope, and the number of cells in each spot. Subsequently, we analyzed gene copy numbers across tumor samples using STARCH. Focusing on mutated genes appearing in the evolutionary tree, we selected genes that were detected across a minimum of 20 tumor spots for both copy number value 1 and 2. This filtering ensured we had sufficient sample sizes for comparison. For each gene we represent 11,461 spots in total. We then plotted the distribution of expression levels (y-axis) grouped by copy number value (x-axis) for these selected genes. The resulting distributions show that genes tending to have higher copy numbers in a tumor sample also exhibit elevated expression compared to genes with lower copy numbers in the same sample.

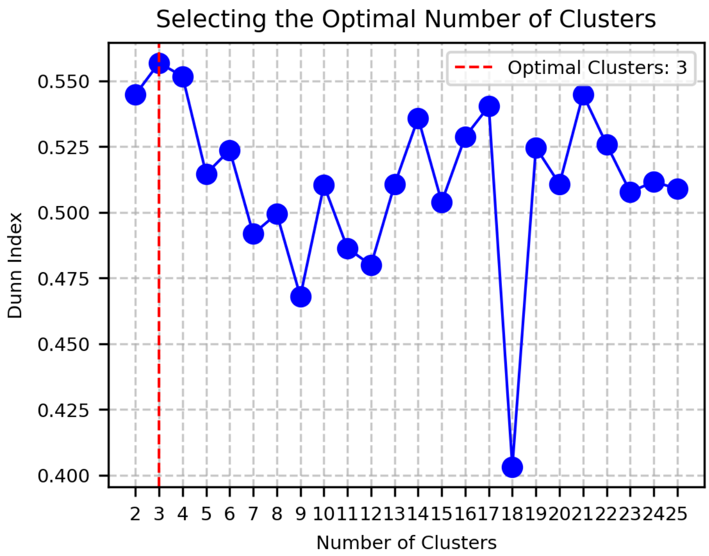

Supplementary Figure 20: Selecting the optimal number of clusters in cell clustering based on scRNA-seq data. y-axis: the Dunn Index score determining the quality of clustering solutions based on scRNA-seq data. The higher the score, the better cluster separation and cohesion. x-axis: the number of clusters in the evaluated clusterings (from 2 to 25).

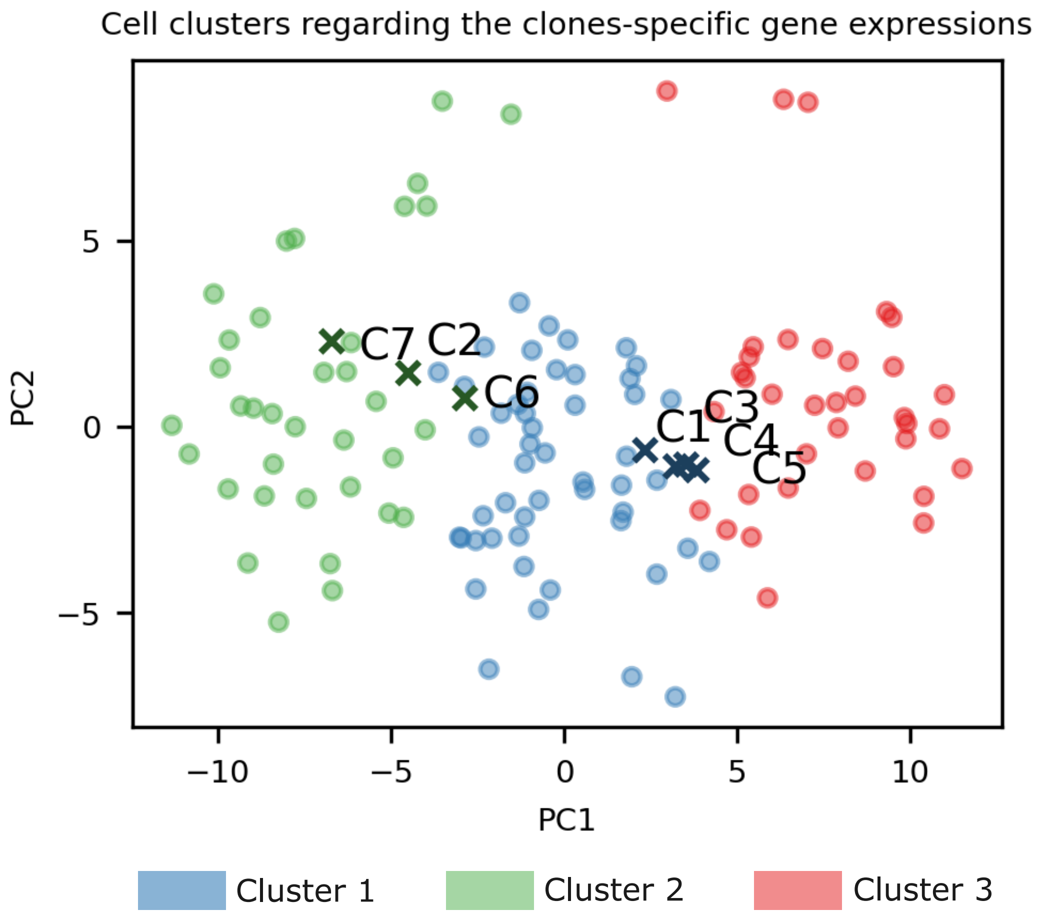

Supplementary Figure 21: Comparison of cell expression profiles in clusters from scRNA-seq and Tumoroscope clone gene expression profiles. Breast cancer cells (depicted by n=120 dots) were collected from the ST sections utilized in this study. They are clustered using K-means based on their normalized expression profiles of the top 100 genes with the highest variance. The gene expression vectors specific to each clone, as determined by Tumoroscope, are visually represented by crosses alongside their respective clone names. These crosses are mapped to the closest centroids and color-coded accordingly. The ability to map inferred gene expression from Tumoroscope to specific clusters from an orthogonal dataset in scRNA-seq highlights the representativeness of Tumoroscope's clone-specific gene expression for cell phenotypes.
